# Prevalence of Self-Reported Diagnosed Cataract and Associated Risk Factors among Elderly South Africans

**DOI:** 10.3390/ijerph14121523

**Published:** 2017-12-06

**Authors:** Nancy Phaswana-Mafuya, Karl Peltzer, Amelia Crampin, Edmund Ahame, Zinhle Sokhela

**Affiliations:** 1 Office of the Deputy Vice Chancellor: Research and Innovation, North West University, Potchefstroom 2520, South Africa; 2 HIV/AIDS/STI/TB Research Programme, Human Sciences Research Council, Pretoria 0002, South Africa; karl.pel@mahidol.ac.th (K.P.); eahame@hsrc.ac.za (E.A.); zsokhela@hsrc.ac.za (Z.S.); 3 Department of Research & Innovation, University of Limpopo, Turfloop 0727, South Africa; 4 London School of Hygiene and Tropical Medicine, University of London, London WC1E 7HT, UK; Mia.Crampin@lshtm.ac.uk; 5 Office of the Deputy Vice Chancellor for Research and Engagement, Nelson Mandela University, Port Elizabeth 6001, South Africa

**Keywords:** age-related cataracts, blindness, inequalities, risk factors, SAGE, South Africa, Sub-Saharan Africa, lower middle income countries

## Abstract

This paper estimates the prevalence of self-reported cataract and associated risk factors among individuals aged ≥50 years in South Africa. Data from a nationally-representative cross-sectional Study on Global AGEing and Adult Health (SAGE) (*N* = 3646) conducted in South Africa from 2007–2008 was analyzed. The primary outcome was self-reported cataract, and exposures included socio-demographics, self-reported co-morbidities, and behavioral factors. Linearized multivariate logistic regression models were used. The weighted prevalence of self-reported diagnosed cataract was 4.4% (95%CI: 3.4–5.8). Prevalence was greater among individuals with advancing age (10.2%), higher quality of life (QoL) (5.9%), education (5.2%), and wealth (5.8%) than their counterparts. Prevalence was also higher among individuals with depression (17.5%), diabetes (13.3%), hypertension (9.1%), and stroke (8.4%) compared to those without these conditions, with the exception of obesity (4.2%). In the final multivariate model, the odds of self-reported cataract were: 4.14 times higher among people ≥70 years than 50 to 59 year olds (95%CI: 2.28–7.50); 2.48 times higher in urban than rural residents (95%CI: 1.25–4.92); 5.16, 2.99, and 1.97 times higher for individuals with depression (95%CI: 1.92–13.86), hypertension (95%CI: 1.60–5.59), and diabetes (95%CI: 1.07–3.61), compared to those without these conditions.

## 1. Introduction

Over 90% of visually impaired and blind people reside in lower middle income countries (LMIC) where health care access is limited [1,2]. Similarly, more than 90% of the estimated global cataract prevalence is experienced in LMIC [3]. Age-related cataracts constitute approximately 50% of the 285 million visually-impaired people globally; 39 million (13.7%) of whom are blind [4]. In Sub-Saharan Africa, they account for 50% of the 26 million visually-impaired people, 5.9 million (22.7%) of whom are blind [5,6]. Over 80% of all blindness is concentrated in individuals aged ≥50 years [7]; a rapidly growing population in LMIC, including South Africa [8,9]. Despite Sub-Saharan Africa (SSA) having one of the highest prevalence of cataracts and blindness, it remains a grossly underserved region [10]. A limited number of population-based studies have been conducted on the prevalence of self-reported cataract in LMIC and found varied prevalence [11,12]. The studies found higher prevalence of self-reported cataract among individuals with older age, lower income, lower education, urban residency, female sex, diabetes, hypertension, and intake of antidepressants and self-reported cataracts [11,12,13]. Inconsistent associations have been found between self-reported cataract, BMI, and lifetime alcohol use and lifetime smoking [11,12]. Eitminam found the prevalence of cataract to be 15% higher among individuals on antidepressants [13]. Rim et al. found extreme stress to be negatively associated with having cataract [11]. Yawson et al. found weak associations between self-reported cataract and quality of life (QoL) [12].

The prevalence of self-reported cataract and its association with the known risk factors among older South Africans is not known, thus, requiring further investigation as the population ages in order to guide future research, prevention strategies, public health policies, and programs in line with Vision 2020 [14].

Data from the Study on Global AGEing and Adult Health (SAGE) were analyzed to examine the prevalence of self-reported cataract and associated socio-demographic (distal) risk factors, co-morbidities, and health behaviours among 3649 South Africans aged 50 years and older.

## 2. Methods

### 2.1. Data Source

The World Health Organization (WHO) conducted a population-based prospective study (SAGE) of people aged 50 years and older in collaboration with research agencies in six LMIC countries (China, Ghana, India, Mexico, Russia, South Africa) from 2007–2010 [15]. This analysis is based on cross-sectional data from SAGE South Africa (Wave 1), conducted between March 2007 to September 2008 by the WHO and Human Sciences Research Council (HSRC). Details on how sampling, enumeration procedures, and post-stratified individual probability weights were conducted to ensure nationally-representative estimates across provinces (strata), geo-type, ethnicity, and sex have been previously published [15,16,17]. This resulted in a multi-stage probability sample of 3649 South Africans aged ≥50 years (73% response rate). Adjusting for the effect of the complex multi-stage cluster random survey design (Design Effect = 1.5), this multistage sample size is equivalent to a simple random sample of 2439 individuals.

### 2.2. Measures

SAGE utilized a validated, pre-tested, and pilot-tested interviewer-administered face-to-face questionnaire [17]. The primary outcome variable of “self-reported cataract” was binary: “In the last five years, were you diagnosed by a health professional with a cataract in one or both of your eyes?” (*Yes or No*).

Exposures were socio-demographics, self-reported co-morbidities and behavioral factors, as described below. Eight socio-demographic characteristics were assessed. Age (in 10-year bands), gender, and geo-locality (urban/rural) had mutually exclusive qualitative categories. Education was coded into four ordered categories: no schooling, <primary, completed primary, and at least secondary. Ethnicity was coded as Black, White, Coloured, and Asian. Wealth quintiles were derived from raw continuous estimates of asset ownership using pure random effects and Bayesian post-estimation models [15,18] and were recoded into a dichotomous variable (quintiles 1,2,3 = low, quintiles 4,5 = high). QoL was assessed using an eight-item WHOQoL internationally-validated tool with two questions taken from each domain: “physical, psychological, social and environmental” [18,19]. The results from the eight items were added up and transformed to obtain total WHOQoL score ranging from 0–100 with lower scores signifying better QoL. This was then coded into ordered categories: low, medium, and high QoL.

Six co-morbidities were assessed by self-report. Amongst these were included questions such as; “Have you ever been diagnosed with stroke, depression, hypertension, and diabetes mellitus?” (*Yes or No*). Obesity (BMI ≥ 30 kg/m^2^) was determined by calculating weight in kilogram (kg) (using a calibrated weighing scale) divided by the square of height in metres (using a stadiometer).

Two health behavior variables were assessed by self-report: “Have you ever smoked (sniffed or chew) tobacco (pipes, snuff, cigars), or consumed a drink that contains alcohol (beer, spirits, wine)?” (*Yes or No*) [15].

### 2.3. Data Analysis

STATA methods [20] were used to find estimates that took into consideration the complex survey design, i.e., weighting, clustering, and stratification. Taylor linearized variance estimation was used. The proportion of missing values for each variable was <5% and records were accordingly excluded from analysis, e.g., all records of cataracts (yes/no) with missing values (175 observations) and answered “don’t know” to cataract questions (16 observations) were excluded from the analysis, resulting in 3649 records for analysis. Linearized logistic regression entailed four stages. Univariate and multivariate analyses were conducted to determine crude and adjusted odds ratios (aOR) for the associations between the exposure variables (socio-demographics, co-morbidities, and health behaviour) and self-reported cataract, respectively, as well as to assess potential confounding of these same variables. The strengths of the associations were determined using a 95%CI and adjusted *p*-values for the Wald test. Multi-collinearity was assessed using a variance inflation factor. Following this, the final model was developed taking statistical efficiency and design effects into consideration. Effect modification by age group was assessed on the final model, using adjusted Wald test (*p* < 0.05).

### 2.4. Ethics Approval

Ethics approval was secured from the HSRC (Reference: 5/13/04/06), WHO (Reference: RPC 149) and London School of Hygiene and Tropical Medicine (reference: 10699) ethical review committees. Approval for the study was also provided by the local National Department of Health (J1/14/45, 2007). Informed consent was secured from each participant in writing prior to each interview.

## 3. Results

### 3.1. Sample Characteristics and Prevalence of Self-Reported Cataract

Sample characteristics and prevalence of self-reported cataract in 3649 participants (73% response rate) are described in Table 1. Most participants reported higher wealth (58.7%); medium/high QoL (93%), and had some schooling (62.7%). Obesity was common (46.8%). Approximately 25.1% ever drank alcohol and 33.4% ever smoked. The weighted prevalence of self-reported cataract was 4.4% (95%CI: 3.4–5.8).

Prevalence of cataract increased with advancing age and was 10.2% in the oldest age category. It was 4.2% among women, 3.2% among blacks, and 2.2% in rural residents, and among individuals with higher QoL (5.9%), secondary plus education category (5.2%), and high wealth category (5.8%). Prevalence of cataract was high among individuals with co-morbidities: depression (17.5%), diabetes (13.3%), hypertension (9.1%), and stroke (8.4%).

### 3.2. Crude Associations between Self-Reported Cataract and Exposures

Table 2 reports the univariate analysis of the factors associated with self-reported cataract (Model 1). The sample included 167 people with self-reported cataract, which was large enough to allow for 16 parameters in the final model. The odds of self-reported cataract were: 4.43 times higher among individuals aged ≥70 years than 50–59 year olds (95%CI: 2.41–8.12; *p* < 0.001); 2.67 times higher among urban dwellers than rural ones (95%CI: 1.42–5.03; *p* = 0.002); 42% lower among individuals in higher than lower wealth category (95%CI: 0.35–0.97; *p* = 0.037); 2.32 times higher among Asians than Blacks (95%CI: 1.07–5.04; *p* = 0.034), and 5.05, 4.17, and 4.13 times higher for individuals with depression, diabetes, and hypertension, respectively, with correspondingly significant 95%CI of 1.97–13.01, 2.31–7.53, and 2.23–7.63) compared to their counterparts.

### 3.3. Final Multivariate Model (Model 4) of Risk Factors for Cataract

The final model that explains how multiple risk factors play a part in self-reported cataract is displayed in Table 3. The odds of self-reported cataract were: 4.14 times higher among 70+ years olds than that of 50–59 year olds (95%CI = 2.28–7.50; *p* < 0.001); 2.48 times higher among urban than rural residents (95%CI = 1.25–4.92; *p* < 0.01); 5.16, 2.99, and 1.97 higher for individuals with depression (95%CI: 1.92–13.86), hypertension (95%CI: 1.60–5.59), and diabetes (95%CI: 1.97–3.61), respectively, compared to their respective reference groups.

## 4. Discussion

The weighted prevalence of self-reported diagnosed cataract among older South Africans was 4.4%, comparable to that of elderly Ghanaians (5.4%) [12], but lower than that of elderly Koreans (11%) [11]. Prevalence in this study relied on participants’ access to health care services, i.e., individuals who were informed by a doctor that they had cataracts. Therefore, the lower prevalence observed may be attributed to undiagnosed cataracts not being included, among socio-economically disadvantaged elderly South Africans who could not access health services due to disparities in availability, accessibility, and affordability of cataract screening services [2,14,21,22,23,24,25,26]. Countries with universal health care systems, like in South Korea, tend to exhibit higher prevalence of self-reported cataract [11].

Consistent with other studies, the multivariate model revealed a strong association between increasing age and prevalence of cataract [11,12]. Cataract development is an age-associated phenomenon [21] and will likely increase further due to the “graying” of the population in South Africa and other LMIC.

Socio-economically disadvantaged groups had significantly relatively lower prevalence of self-reported cataract, i.e., female, black, rural residents and individuals from lower educational backgrounds, QoL, and wealth groups, contrary to other studies [11,12,22,23].

The lower prevalence of diagnosed cataracts among socio-economically disadvantaged groups in South Africa could be attributed to a range of socio-economic vulnerabilities leading to underutilization of and unequal access to cataract (screening) services and, consequently, blindness [24]. This may include limited mobility due to the lack of eye-care resources, dependence on ad hoc eye care outreach services, lack of transportation fare to eye care facilities which may only be in large cities, and long waiting periods.

This is unlike other studies where individuals with higher socio-economic status reported to have a lower prevalence of cataract [11,22].

Health inequalities persist in RSA [27] in spite of improvements in health access [28,29]. Cataracts co-exist with diabetes and hypertension in this analysis, consistent with other studies [11,12,22,23,30]. The biological plausibility of the association between the two conditions (“sugar-cataracts”) has long been documented [31,32]. The combined impact of cataract and these highly-prevalent co-morbid conditions in the sample (diabetes—9.1%; hypertension—30.2%) has serious psycho-social, health care, and service delivery implications in terms of increased mortality, morbidity, demand for health care services, long-term care, health care expenditure, and health care budgets [16,33,34,35,36,37]. The findings call for regular eye examination among diabetics and people with hypertension.

The study also found the prevalence of self-reported cataract to be higher among depressed individuals (17.5%) than non-depressed ones (4%); depressed individuals were 2.97–4.18 times more likely to have self-reported cataract than non-depressed ones. Although one study [13] found the odds of cataract to be 15% higher among individuals on antidepressants, causality could not be confirmed in the current study due to cross-sectional design. 

Prospective studies are needed to have a more in-depth understanding of the association between cataract and depression, as well as to examine reverse causation [38,39,40,41,42,43,44].

It is unclear why there was lower prevalence of self-reported cataract among obese (4.2%) compared to non-obese (4.5%) individuals and why obese individuals were 13–26% less likely to have cataract than non-obese ones in multivariate analysis, given known biological plausibility, i.e., physiological mechanisms (oxidative stress, metabolic syndrome, insulin resistance, glycosylation, and uric acid concentrations) that facilitate cataract development and other chronic diseases [45,46]. Similar results were also reported in another cross-sectional study, but it was unclear how this came about [47]. The possibility of reverse causality cannot be ruled out. It is possible that individuals in this study may have developed cataract before they became obese. SAGE follow-up data analysis is needed to determine causality. These findings may also be confounded by unmeasured confounders (nutritional status, physical activity, etc.).

The prevalence of self-reported cataract was higher among individuals with prior stroke (8.4%) compared to those without it (4.2%), as in other studies [12]. Stroke sufferers were 2.06 times more likely to have cataract than non-sufferers. Stroke and cataract are generally co-morbidities due to similar aetiologies and risk factors (oxidative stress, atherosclerosis, age, diabetes, hypertension, and trauma) [48]. The low prevalence of cataract among lifetime smokers (3.8%) and drinkers (3.7%) is contrary to other studies [12,49], as lifetime smokers were 13–26% less likely to have cataract than smokers. Perhaps individuals with cataracts underreported lifetime tobacco use as they may have thought that they may be accused of bringing cataracts upon themselves through smoking. It may also be due to low levels of smoking; individuals who participate in health research may be moderate smokers and, thus, have a lower risk of oxidative stress and subsequently cataract. The fact that individuals who drank alcohol were more likely to have cataract is in line with other studies [12]. High alcohol intake increases the risk of vitamin deficiencies, membrane damage, and disruption of calcium homeostasis responsible for cataract formation [3,50,51].

## 5. Conclusions

The estimated prevalence of self-reported cataract was low; this is likely due to undiagnosed cataract which was not considered. Lower SES groups had lower prevalence while higher SES groups had higher prevalence. This may reflect socio-economic disparities in the availability, accessibility, and affordability of eye care services which makes cataracts appear like a disease of the rich, masculine, and non-black people. Basing planning, prevention and intervention efforts on the burden of cataract reported here may perpetuate inequities. Prioritization of cataract services and interventions to socio-economically disadvantaged individuals is critical. Age and being a rural resident, as well as diabetes, hypertension, and depression were risk factors for self-reported cataract. However, the results in this regard are not conclusive given the cross-sectional nature of the study design.

Future studies should estimate the prevalence of both previously diagnosed and undiagnosed cataract in order to have a complete picture of the burden of cataracts among elderly South Africans. SAGE follow-up surveys may determine new cases of cataract and the causality of the associations found here. It is also essential to develop a comprehensive social protection strategy such as the National Health Insurance System (NHIS) in order to ensure the provision of adequate, effective, ongoing, equitable, accessible, and affordable health services in line with the RSA Constitution and, thus, contribute to the realization of Vision 2020.

## Figures and Tables

**Table 1:** Sample characteristics and prevalence of self-reported cataract.

Explanatory Factors	Sample Characteristics		Prevalence of Self-Reported Cataract	
**Socio-Demographics**	**Sample *N* = 3649**	**Weighted %** **(95%CI)**	**No. of People with Cataract *N* = 167**	* Weighted % **(95%CI): 4.4 (3.4–5.8)**
*Age (years)*				
50–59	1607	49.8 (46.8–52.9)	40	2.5 (1.5–4.0)
60–69	1176	30.8 (28.5–33.3)	55	3.9 (2.6–5.8)
≥70	866	19.4 (17.3–21.7)	72	10.2 (6.9–14.8)
*Gender*				
Male	1551	43.9 (40.9–47.0)	64	4.7 (3.1–7.1)
Female	2098	56.1 (53.0–59.1)	103	4.2 (3.0–5.8)
*Ethnicity*				
Black	1975	61.3 (56.8–65.5)	74	3.2 (2.4–4.5)
White	255	7.7 (5.5–10.8)	17	6.4 (2.5–15.5)
Coloured	647	10.9 (8.7–13.6)	37	5.8 (3.1–10.5)
Asian	287	3.2 (2.3–4.3)	19	7.2 (3.6–14.0)
*Education*				
No schooling	1331	37.2 (33.9–40.6)	44	4.1 (2.6–6.4)
Incomplete Primary	784	20.8 (18.3–23.6)	39	3.5 (2.1–5.8)
Completed Primary	742	18.4 (16.2–20.9)	37	5.1 (3.1–8.4)
Secondary plus	792	23.5 (20.6–26.8)	47	5.2 (3.2–8.5)
*Wealth*				
Low	1520	41.3 (36.6–46.2)	93	3.4 (2.4–5.0)
High	2111	58.7 (53.8–63.5)	72	5.8 (4.1–8.1)
*Geo-locality*				
Rural	1203	65.1 (59.2–70.6)	35	2.2 (1.3–3.7)
Urban	2443	34.9 (29.4–40.8)	131	5.6 (4.2–7.5)
*Quality of Life*				
Low	209	7.0 (5.5–8.7)	8	2.3 (0.9–6.0)
Medium	2325	64.4 (60.9–67.8)	106	4.0 (2.9–5.6)
High	1078	28.6 (25.2–32.3)	53	5.9 (3.4–5.8)
**Co-morbidities**				
*Stroke*				
Yes	139	4.0 (3.0–5.4)	11	8.4 (3.7–17.7)
No	3510	96.0 (94.6–97.0)	156	4.2 (3.2–5.6)
*Hypertension*				
Yes	1115	30.2 (27.1–33.5)	58	9.1 (6.7–12.3)
No	2534	69.8 (66.5–72.9)	109	2.4 (1.4–3.9)
*Depression*				
Yes	113	2.9 (2.1–3.9)	13	17.5 (7.9–34.4)
No	3536	97.1 (96.1–97.9)	154	4.0 (3.0–5.3)
*Diabetes*				
Yes	358	9.1 (7.6–10.7)	45	13.3 (8.2–20.6)
No	3291	90.9 (89.3–92.4)	122	3.5 (2.6–4.8)
*Obesity*				
Yes	1519	46.8 (43.3–50.4)	77	4.2 (3.1–5.8)
No	2052	52.1 (48.5–55.7)	87	4.5 (3.1–6.7)
**Health behaviours**				
*Alcohol use (ever)*				
Yes	996	25.1 (22.5–28.0)	45	3.7 (2.5–5.5)
No	2653	74.9 (72.0–77.6)	122	4.7 (3.4–6.3)
*Tobacco use (ever)*				
Yes	1294	33.4 (30.5–36.5)	55	3.8 (2.4–6.1)
No	2355	66.6 (63.5–69.5)	112	4.7 (3.5–6.4)

* The percentages were weighted to the general South African population.

**Table 2:** Crude associations between self-reported cataract and exposures (Model 1).

Socio-Demographics	OR (95%CI)	Wald Test *p*-Value	* Adjusted Wald Test *p*-Value
*Age (years)*			0.000 **
50–59	1.00		
60–69	1.58 (0.85–2.93)	0.147	
70+	4.43 (2.41–8.12)	0.000	
*Gender*			
Female	1.00		
Male	1.13 (0.65–1.96)	0.669	0.669
*Ethnicity*			0.063 ***
Black	1.00		
White	2.05 (0.73–5.78)	0.172	
Coloured	1.84 (0.89–3.81)	0.099	
Asian	2.32 (1.07–5.04)	0.034	
Education ****			0.656
No schooling	1.00		
<primary	0.86 (0.45–1.64)	0.642	
Primary	1.28 (0.63–2.61)	0.493	
Secondary plus	1.30 (0.67–2.53)	0.437	
*Wealth*			0.037 **
High	1.00		
Low	0.58 (0.35–0.97)	0.037	
*Geo-locality*			0.002 **
Rural	1.00		
Urban	2.67 (1.42–5.03)	0.002	
*Quality of Life*			0.145
Low	1.00		
Medium	1.75 (0.64–4.78)	0.277	
High	2.63 (0.93–7.47)	0.068	
Co-morbidities			
*Stroke*			0.099 ***
No	1.00		
Yes	2.06 (0.87–4.85)	0.099	
*Hypertension*			0.000 **
No	1.00		
Yes	4.13 (2.23–7.63)	0.000	
*Depression*			0.001 **
No	1.00		
Yes	5.05 (1.97–13.01)	0.001	
*Diabetes*			0.000 **
No	1.00		
Yes	4.17 (2.31–7.53)	0.000	
Obesity ****			0.780
No	1.00		
Yes	0.93 (0.56–1.55)	0.780	
Health Behaviours			
Alcohol use (ever) ****			0.358
No	1.00		
Yes	0.79 (0.48–1.31)	0.358	
Tobacco use (ever) ****			0.453
No	1.00		
Yes	0.81 (0.46–1.41)	0.453	

NB: The first category of each variable is reference group; * Overall association between each explanatory variable and cataract; ** Strong evidence of association; *** Some weak or marginal evidence of association; **** A priori factors.

**Table 3:** Final multivariate model of risk factors for self-reported cataract.

Socio-Demographics	Adjusted OR (95%CI)	*p*-Value	* Adjusted Wald Test *p*-Value
*Age (years)*			0.000 **
50–59	1		
60–69	1.24 (0.65–2.35)	0.515	
70+	4.14 (2.28–7.50)	0.000	
*Education*			0.846
No schooling	1.00		
<Primary	0.76 (0.39–1.49)	0.418	
Primary	1.02 (0.49–2.12)	0.956	
Secondary plus	1.00 (0.52–1.90)	0.994	
*Wealth*			0.744 ****
High	1.00		
Low	0.91 (0.53–1.59)	0.744	
*Geo-locality*			0.010 **
Rural	1.00		
Urban	2.48 (1.25–4.92)	0.010	
Co-morbidities			
*Hypertension*			0.001 **
No	1.00		
Yes	2.99 (1.60–5.59)	0.001	
*Depression*			0.001 **
No	1.00		
Yes	5.16 (1.92–13.86)	0.001	
*Diabetes*			0.029 **
No	1.00		
Yes	1.97 (1.07–3.61)	0.029	
*Obesity*			0.448 ****
No	1.00		
Yes	1.25 (0.71–2.20)	0.448	
Health Behaviours			
*Lifetime alcohol (ever)*			0.942 ****
No	1.00		
Yes	1.03 (0.50–2.11)	0.942	
*Tobacco use (ever)*			0.665 ****
No	1.00		
Yes	0.85 (0.42–1.73)	0.665	

NB: The first category of each is reference group; * Assesses overall association between each explanatory variable and self-reported cataract; ** Strong evidence of association; **** A priori factors.
